# Chimeric Structures in Mental Illnesses—“Magic” Molecules Specified for Complex Disorders

**DOI:** 10.3390/ijms23073739

**Published:** 2022-03-29

**Authors:** Patrycja Kleczkowska

**Affiliations:** 1 Maria Sklodowska-Curie Medical Academy in Warsaw, Solidarnosci 12 Str., 03-411 Warsaw, Poland; hazufiel@wp.pl; Tel.: +48-690-888-774; 2 Military Institute of Hygiene and Epidemiology, 4 Kozielska Str., 01-163 Warsaw, Poland

**Keywords:** chimeric compounds, mental disorders, schizophrenia, depression, efficacy

## Abstract

Mental health problems cover a wide spectrum of diseases, including mild to moderate anxiety, depression, alcohol/drug use disorders, as well as bipolar disorder and schizophrenia. Pharmacological treatment seems to be one of the most effective opportunities to recover function efficiently and satisfactorily. However, such disorders are complex as several target points are involved. This results in a necessity to combine different types of drugs to obtain the necessary therapeutic goals. There is a need to develop safer and more effective drugs. Considering that mental illnesses share multifactorial processes, the paradigm of one treatment with multiple modes of action rather than single-target strategies would be more effective for successful therapies. Therefore, hybrid molecules that combine two pharmacophores in one entity show promise, as they possess the desired therapeutic index with a small off-target risk. This review aims to provide information on chimeric structures designed for mental disorder therapy (i.e., schizophrenia and depression), and new types of drug candidates currently being tested. In addition, a discussion on some benefits and limitations of multifunctional, bivalent drug candidates is also given.

## 1. Introduction

Mental disorders constitute a wide range of health conditions associated with alterations in mood and/or thinking, which consequently affect the way we act and behave. The most common mental disorders that currently burden numerous countries include depression, bipolar disorder, schizophrenia, and obsessive–compulsive disorder (OCD). However, according to the Diagnostic and Statistical Manual of Mental Disorders (DSM-5), there are several other pathological states. The DSM-5 is now a reliable source for clinicians to help them make more accurate diagnoses and improve patient outcomes, particularly due to the necessity to consider the relevance of age, gender, and culture. Nevertheless, mental disorders rank third among the global most frequent diseases after cancer and cardiovascular diseases [1].

Surprisingly, a variety of mental illnesses appear in young individuals, though the level of prevalence among these groups of patients varies greatly in different parts of the world, ranging from 3.5 to 40.3% [2,3]. Both retrospective and prospective research revealed that most adulthood mental disorders begin in childhood and adolescence [4]. Indeed, it is suggested that the development of psychotic disorders (at least some) may result from a so-called “difficult childhood”, which includes being raised in a pathological, disrupted, or dysfunctional family, with an atmosphere of either lack of love or emotional coldness. The likelihood of illness also increases in those who have had a traumatic experience such as the death of a loved one, an accident, or sexual violence (rape/sexual assault) [5]. Genetic issues were also considered linked to psychiatric disorders [6]. In fact, Birmaher et al. [7] claimed that twin and adoption studies have shown that genetic factors account for at least 50% of the variance in the transmission of child and adolescent mood disorders in those whose parent(s) also suffered from some kind of mental illness; however, this was not confirmed by others [8].

The occurrence of mental illnesses may also be a consequence of the chronic use of psychoactive substances (e.g., alcohol, narcotic drugs, but—surprisingly—not nicotine), or altered serotonin activity [9,10]. Paradoxically, some mental illnesses may lead to substance abuse, because of self-medication, and thus—ultimately—broadening the spectrum of the central nervous system (CNS) damage.

Unfortunately, mental disorders have a significant impact on quality of life. Indeed, people suffering from depression or other serious psychiatric disorders face prejudice and discrimination and are stigmatized. These people can sometimes represent a danger to the public, which results in social exclusion and self-esteem reduction. Furthermore, for many sufferers, suicide is the only way to defeat the “enemy within”. In fact, the risk of death among mentally ill individuals is several times higher than in the rest of the population [11].

Most of the illnesses affecting our mental health are shown to have a highly complex molecular background. Currently available treatments can provide symptomatic, temporary relief for a limited time or may have no impact at all. Additionally, what is good for one individual may not be effective for everyone. Importantly, it is known that many people may suffer from more than one condition and therefore mix several drugs. This, in turn, increases the risk of burdensome and clinically significant side effects resulting from drug–drug interactions.

Given the multi-target background of mental health disorders, various ligands possessing different activities (e.g., *N*-methyl-D-aspartate (NMDA) and dopamine (DA) receptors in schizophrenia) within a single molecule seem to be a proper solution. 

This paper focuses on some of the most common psychiatric disorders, together with the characterization of new types of investigational compounds and drug candidates currently being tested. In addition, a discussion regarding some of the benefits and limitations of multifunctional bivalent drug candidates is provided. 

### Hybrid Approach in Drug Discovery and Development

**Figure 1:**
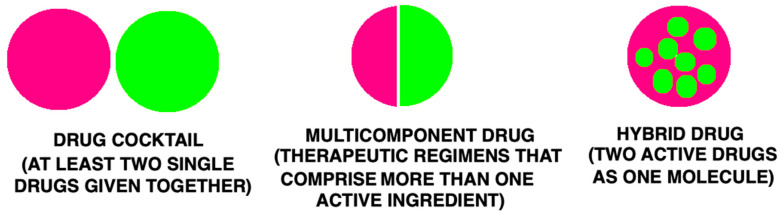
Types of multitarget therapies.

Patients suffering from various diseases, especially those characterized by high complexity, often use several drugs simultaneously. Sometimes, this necessity to co-administer different active substances at one time results from diverse kinds of pathological states, e.g., obesity and diabetes, pain, and cancer. Currently, three types of polytherapy can be distinguished (Figure 1). Firstly, single drugs may be given together as a drug cocktail. Secondly, multicomponent drugs comprising more than one active ingredient may be administered. Finally, drugs can be applied as chimeras (hybrids), i.e., a single structure comprises pharmacophoric moieties of known molecules (with two or more structural domains) [12]. 

On the one hand, polypharmacotherapy intensifies the effectiveness of treatment due to possible interactions with multiple targets, improving the response by multidirectional action; on the other, the use of single or multicomponent drugs simultaneously may result in fatal adverse effects. These are related to pharmacokinetics and/or the pharmacodynamics of drug–drug interactions.

For instance, the use of two active substances in one tablet (a so-called multicomponent drug), though being an attempt to alleviate possible interactions, does not resolve the problem. However, such refinement of therapy by reducing the number of medications improves so-called “compliance”—the cooperation between the patient and the doctor [13]. It seems to be extremely important, especially for patients who do not fully follow their doctors’ recommendations. However, ultimately, the therapeutic effectiveness of this type of drug remains under question.

Given the limitations of the traditional paradigm of “one drug—one goal (target)—one disease”, especially in the complex nature of many psychiatric disorders, either the multifunctionality of hybrid compounds or the hybrid drug discovery approach may represent a promising alternative. 

**Figure 2:**
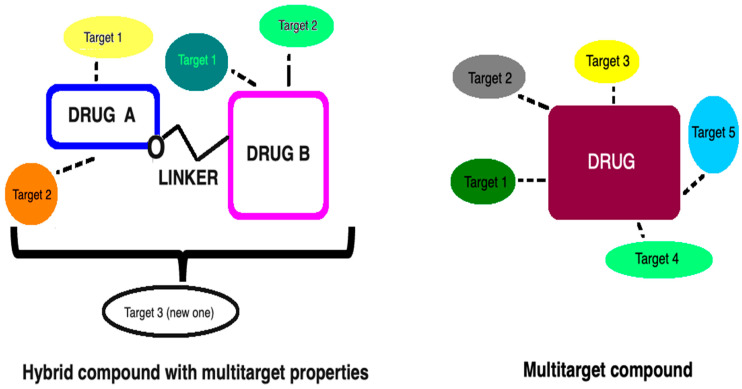
Comparison of multitarget compounds vs. hybrid compounds with multitarget properties. Multitarget compounds that are not bivalent hybrid molecules do not possess specific building fragments corresponding to original drugs or other known ligands. On the other hand, they are characterized by having recognized and privileged small fragments/regions that are able to target different systems (e.g., heterocyclic cores, aliphatic residues). In contrast, hybrid compounds encompass one or more bioactive compounds from a different class or their pharmacophoric subunits (drug A, drug B) into one molecule, thereby representing the desired features of the original drugs. In the figure, the hybrid structure is built on the basis of a conjugation of pharmacophores connected via any cleavable linker (e.g., esters, amides, carbamates, or disulfide bonds).

**Figure 3:**
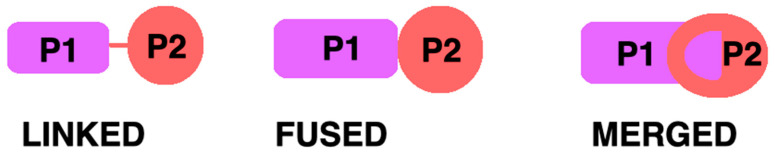
Classification of hybrid ligands (chimeras) based on pharmacophore (P) linking.

In fact, bivalent drugs (known as hybrids, chimeras, designed multiple ligands (DMLs)), in contrast to popular multicomponent drugs, constitute one chemical molecule. They combine various active elements, being clinically available drugs or structures naturally occurring in our body; thus, additional multifunctionality exists (Figure 2). However, for the multitarget drugs that are not hybrid molecules, the identification of a molecule’s structural element that is responsible for a given effect or interaction with a specific molecular target is extremely difficult. Nevertheless, the components (pharmacophores (P)) of such hybrid compounds can be combined using one of the three basic types of coupling (Figure 3). This, in turn, appears to be the desired way to balance the unwanted side effects derived from their components. 

**Figure 4:**
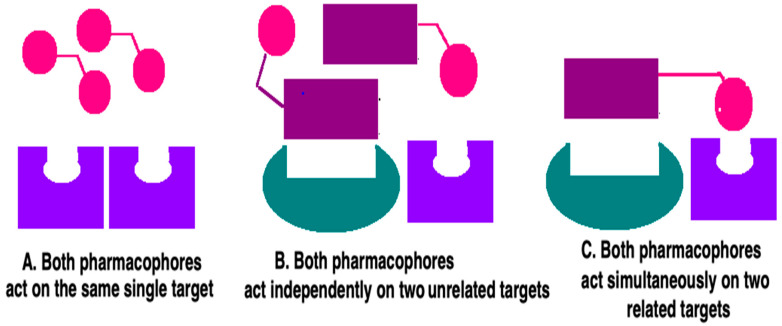
Mode of hybrid drugs related to target interactions.

Thus, these new molecules can be the proper solution for many patients suffering from one type of disease with multifactorial etiology, as they give the opportunity to simultaneously influence several important mechanisms involved in the molecular background of a given disease. Furthermore, they may prove effective in the treatment of various diseases occurring simultaneously [14,15]. This unique property occurs due to the ability of this type of structure to interact with its target(s) in several different ways (Figure 4). If a given disease concerns a single target, then it is assumed that both hybrid pharmacophores can act with a target (Figure 4A). In contrast, in the case of having two diverse targets (Figure 4B), the two entities of the hybrid molecule may then act independently on these two different and unrelated receptors (or enzymes, etc.). The third possible way of action is based on targeting two related/connected targets using both entities (pharmacophores) (Figure 4C).

Due to their specific and unique structure, apart from the aforementioned properties, such compounds may have significantly improved and more predictable pharmacokinetics and pharmacodynamics, including increased resistance to numerous enzymes [12]. These types of compounds possess the ability to penetrate several physiological CNS barriers, including the blood–brain barrier (BBB) [16,17], although according to Lipinski’s Rule of 5, the solubility and membrane permeability may be affected due to the increased molecular weight. Surprisingly, their molecular weight does not limit their therapeutic usefulness. In systemic-based therapies, most of such compounds’ molecular weight is estimated at >500 Da, yet they can still be characterized by—at least minor—BBB permeability. Intriguingly, such a property is seen for hybrid compounds composed of fragments that have negligible ability to pass across this highly selective barrier [16,18]. Therefore, a completely new activity is observed that cannot be predicted solely from the structural elements of the molecule. Additionally, according to some evidence available in the literature, hybrid compounds exhibit an improved safety profile much in line with our requirements, compared to polytherapy composed of individual chimeric components (pharmacophores) [18].

Although hybrid drugs overcome a long list of disadvantages manifested by single-target drugs or still-popular polytherapy, they may also have some disadvantages. One of the major problems concerns difficulties in designing compounds with balanced activity to multiple targets. Thus, there is no possibility to conjugate two moieties of interest at specific concentrations or doses, as the ratio of the respective activities cannot be titrated. More clearly, doses cannot be as flexibly administered as in the case of single drugs. This, in turn, can be problematic and challenging in terms of choosing the right combination of targets for the disease of interest. In addition, the possible effects of a synergistic, additive, or inhibitory nature should be considered when modulating the selected targets. Of note, additive effects can be obtained when the targets belong to the same pathway, whereas synergism can only be achieved for selected targets located on functionally complementary pathways [19].

Moreover, as mentioned previously, in the case of such new types of structures, the prediction of their biological activities based solely on the activities of each hybrid’s structural pharmacophore can sometimes be incorrect. In fact, the molecule as a whole is characterized by a completely different biological activity compared to its components given alone. Consequently, it may interact with additional targets not included in the design process [20]. Finally, chimeric drugs, especially being peptides, may possess relatively low bioavailability, which might be further associated with their short half-life or high sensitivity to proteolytic enzymes. In addition, such compounds usually have negligible ability to generate a central pharmacological effect after peripheral administration, particularly as a consequence of their high molecular weight. However, these parameters are not constant, because a number of methods have been developed to improve the aforementioned properties without affecting drug biological activity (e.g., by replacing the amide bonds with bioisosteric equivalents, cyclization of the peptide chain, or creating conjugates with polymers) [21].

## 2. Psychiatric Disorders and the Usefulness of Bivalent Drugs

Numerous psychiatric disorders are characterized by possessing complex pathomechanisms. Unfortunately, single-target drugs that are commercially available and somehow efficacious in reducing some symptoms of the disease may not provide the desired effect. Obviously, they are able to act on the target of interest, but when co-administered with other drugs with a different mechanism of action, are likely to lead to pharmacodynamic and pharmacokinetic interactions. Moreover, in long-term care, they may also result in various side effects, which usually discourage patients from taking their medications. Therefore, a hybrid approach seems to be a perfect way to balance the unwanted side effects derived from individual components.

Great effort and progress were made over the last decades to design and develop drug candidates with dual activity that may be useful in diseases/disorders where multiple pathomechanisms are observed.

### 2.1. Schizophrenia as the Most Prevalent Psychotic Disorder

#### 2.1.1. Etiology of Schizophrenia and Currently Available Drugs

Schizophrenia is a mental illness affecting 0.5–1% of the world’s population, mostly occurring in late puberty and early adulthood. It disrupts normal brain functioning in specific brain regions such as the hippocampus and prefrontal cortex (PFC), resulting in distinct symptoms that can obscure both personality and intellect and make individuals unable to pursue an independent life. There are two groups of symptoms: positive (i.e., delusions, hallucinations, and thinking disorders) as well as negative (i.e., social withdrawal, anhedonia) and cognitive deficits (related to attention, working memory, and executive function) [22,23].

Current medical treatment for schizophrenia only addresses the symptoms, because the etiology of the disease remains unknown. Several hypotheses of the possible pathways for schizophrenia are presented below.

***Glutamate and NMDA hypothesis***

The predominant hypothesis for the pathophysiology of schizophrenia is related to the involvement of glutamatergic transmission. Indeed, glutamate (Glu), a major excitatory neurotransmitter in the CNS, was indicated to play a crucial role in normal physiological processes. Consequently, its dysfunction and/or dysregulation leads to the occurrence of profound effects in the wide spectra of diseases. 

In relation to schizophrenia, numerous studies support the idea of Glu dysfunction, starting from the altered expression of different molecules (especially astrocytes [24]) involved in Glu synthesis, transport, binding, re-uptake, and recycling [25,26]; this results in a decrease of ionotropic NMDA receptor activity and reduces the level of Glu in the brain and cerebrospinal fluid [27]. Moreover, the statement that the NMDA receptor blockade plays a pivotal role in the development of the disease was based on studies using phencyclidine (PCP) and other dissociative anesthetic-type psychotomimetics (e.g., ketamine, MK-801), that are NMDA receptor, competitive antagonists, as these drugs were found to induce psychotic symptoms and neurocognitive disturbances similar to those of schizophrenia [28]. Supporting findings have also been driven from genetic studies. In fact, several de novo copy-number variants (CNVs) encoding NMDA receptors together with other proteins associated with the increased risk of schizophrenia were revealed [29,30]. Importantly, apart from the NMDA receptor hypofunction as a process related to schizophrenia, other ionotropic Glu receptors (alpha-amino-3-hydroxy-5-methyl-4-isoazolepropionic acid, AMPA, and kainate receptors) as well as metabotropic Glu receptors were also found to be involved.

***Dopamine-related hypothesis***

The second hypothesis links the schizophrenia mechanism to dopaminergic signaling, which in contrast to the glutamate-based theory, primarily explains the positive schizophrenic symptoms [31]. The model of a presynaptic striatal hyperdopaminergic state and dopamine (DA) receptors excitation was supported by various drug effects. DA agonists such as the psychostimulant amphetamine and PCP may provoke symptoms that resemble those of schizophrenia in some healthy individuals [32,33,34]. In contrast, imaging studies in patients with schizophrenia have shown a deficiency of cortical DA, which seems to be crucial for the negative and cognitive symptoms of schizophrenia [35]. While both the ventral and dorsal striata have shown increased DA release, the magnitude was observed in the nigrostriatal pathway to the dorsal striatum, particularly in the rostral caudate [36]. 

Importantly, the presented findings were confirmed by the reduction in positive symptoms with D2 antagonist therapy [37]. Therefore, in line with this and considering the abovementioned action of PCP on different brain systems, it is strongly suggested that NMDA dysfunction may ultimately lead to a secondary dopaminergic dysregulation in both the striatal and prefrontal brain regions.

***Serotonin and 5HT receptors hypothesis***

The physiology of schizophrenia has expanded beyond both the DA and NMDA function alterations to other hypotheses that include, in particular, serotonin. The first suggestion on the possible involvement of the serotoninergic system in the pathophysiology of this disorder was several decades ago with the examination of LSD-induced psychosis [38].

Currently, several selective and/or potent serotoninergic 5HT antagonists resulted in therapeutic efficacy by reducing the number and intensity of both negative and affective symptoms, mainly mood disturbances [39,40]. Some of them possess additional activity towards the dopaminergic system (i.e., risperidone), by causing a slight, indirect activation of midbrain DA neurons with enhanced DA release in terminal regions. This, in turn, may be associated with the fact that 5HT receptor subtypes (i.e., 5HT_2C_ receptors) were found localized in DA and GABA neurons in the ventral tegmental area (VTA) [41]. Similarly, functional crosstalk between 5HT_2A_ receptors and metabotropic Glu type 2 (mGlu2) receptors was proposed, as both can assemble into a functional heteromeric complex and thus modulate each other’s function.

With regard to schizophrenia treatment, the possible efficacy of serotonin antagonists in this type of mental disorder was further confirmed by several investigations with agonists. In fact, for instance, the 5HT_2C_ receptor agonist CP809101 was demonstrated to up-regulate the performance on some cognitive tasks in animals with decreased 5HT synthesis [42].

Although substantial reports suggest that either massive increases of 5HT availability in the brain exert psychogenic effects, or multiple 5HT receptor dysfunctions are somehow associated with schizophrenia, some bodies of evidence show inconsistent, even contradictory, findings. Therefore, the serotonin involvement in schizophrenia is still under deep investigation.

***Other hypotheses***

Acetylcholinergic system dysfunction is also implicated in the pathophysiology of schizophrenia; this applies primarily to acetylcholine (ACh). Indeed, reduced α7 nAChR expression, having a high affinity for nicotine, has been observed in the hippocampus and cingulate cortex of postmortem brains from schizophrenic patients. In line with this, the stimulation of these receptors in PFC was found to modulate glutamatergic signaling through NMDA receptors by influencing circuits important for working memory performance, although α4β2-nAChRs were suggested to be even more prominent in this respect [43,44]. Another strong argument for the involvement of the ACh system in schizophrenia is based on the fact that smoking alleviates cognitive deficits in schizophrenic patients. Thus, nicotine and other ACh agonists can be crucial in normalizing the brain deficits associated with this disorder. 

Surprisingly, it is postulated that the background of schizophrenia may also be associated with the inflammatory process of the CNS [45]. Microglia seem to be altered (increased) in schizophrenic subjects compared to healthy ones [46,47]. This was also proposed by several authors showing an increased expression of inflammatory cytokines in mRNA (i.e., IL-6, IL-1β, IL-8, TNFα) in the dorsolateral prefrontal cortex in unmedicated schizophrenia patients [48,49]. Moreover, it was found that severe infections and autoimmune disorders additively increase the risk of schizophrenia and schizophrenia spectrum disorders [50,51,52]. The confirmation of the role of the inflammatory process in the development of schizophrenia is the fact that nonsteroidal anti-inflammatory drugs (NSAIDs), such as celecoxib and acetylsalicylic acid, have shown positive effects in schizophrenia spectrum disorders, including the alleviation of both positive and negative symptoms [53,54].

The next hypothesis concerns sex steroid hormone dysfunction, particularly estrogen, as it may affect mood, cognition, and behavior [55]. Concerning schizophrenia, it was reported that most women with this disorder are also hypoestrogenic. In line with this, Kulkarni and colleagues indicated that women with schizophrenia receiving adjunctive estradiol resulted in significant improvements in the positive symptoms and general psychopathology (PANSS) rating scale [56]. To support the possible role of steroid hormones in the development and possible treatment of schizophrenia, a large number of data have shown estrogen’s ability to modulate the numerous neurotransmitter DA, serotonin, and Glu systems [57,58,59].

##### Drugs Clinically Available in the Treatment of Schizophrenia-like Symptoms

There is a growing interest in searching and developing potentially effective drugs in psychotic disorder therapy, though several clinically available effective antipsychotic drugs exist on the market. Indeed, among the most popular antipsychotic drugs, the main groups can be distinguished as: (i) traditional “typical” (e.g., haloperidol, chlorpromazine), and (ii) a new generation of “atypical” drugs (e.g., clozapine, risperidone). While the first group acts on the positive symptoms, the second is generally considered to be effective against both positive and negative symptoms. In contrast to the typical group, atypical drugs have little or no propensity for producing extrapyramidal symptoms (EPS). However, ultimately, most of these drugs still have a risk of metabolic syndrome, weight gain, and hyperprolactinemia [22,23]. Furthermore, such drugs can make psychotic symptoms worse and can lead to sudden cardiac death [60].

Of interest, atypical neuroleptics are multitargeting, as they interact with several various and distinct receptor systems (Figure 4). The goal strategy in schizophrenia treatment consists of blocking dopamine D2, serotonin 5HT_2A_, and α1-adrenergic receptors. Considering this, a great example of such an atypical drug is aripiprazole acting either on the dopamine D2 receptors or the serotoninergic receptors, in particular 5HT_2A_ and 5HT_1A_. Unfortunately, these “fix-all” drugs, despite possessing the ability to interact with targets suggested to be crucial for schizophrenia development, do not remain hybrid drugs. In fact, when we consider agents with a hybrid-based structure, acting on the same or separate targets with a different mechanism of action, only a few meet those criteria (see Section 2.1.2).

The most valuable and popular drugs used in schizophrenia treatment are listed in Table 1.

#### 2.1.2. Multifunctional Chimeras Potent in Attenuation/Reduction of Schizophrenic-like Symptoms

Hybrid compounds are designed to serve as multitarget ligands and are intended for diseases/disorders of high complexity and a multifactorial nature, or to be used as an alternative to polytherapy. However, conversely, multitarget structures are not necessarily chimeras formed by the participation of two (or more) pharmacophore groups. This misunderstanding of hybrid drug definitions is commonly observed in papers also aimed at describing antipsychotic drugs. Indeed, clozapine and other popular antipsychotics were recently presented as multifunctional hybrid molecules, probably due to their ability to interact with several different systems of the CNS. Nevertheless, both neuroleptic-like drug candidates and clinically available neuroleptics, being hybrids, are currently a limited group.

**Figure 5:**
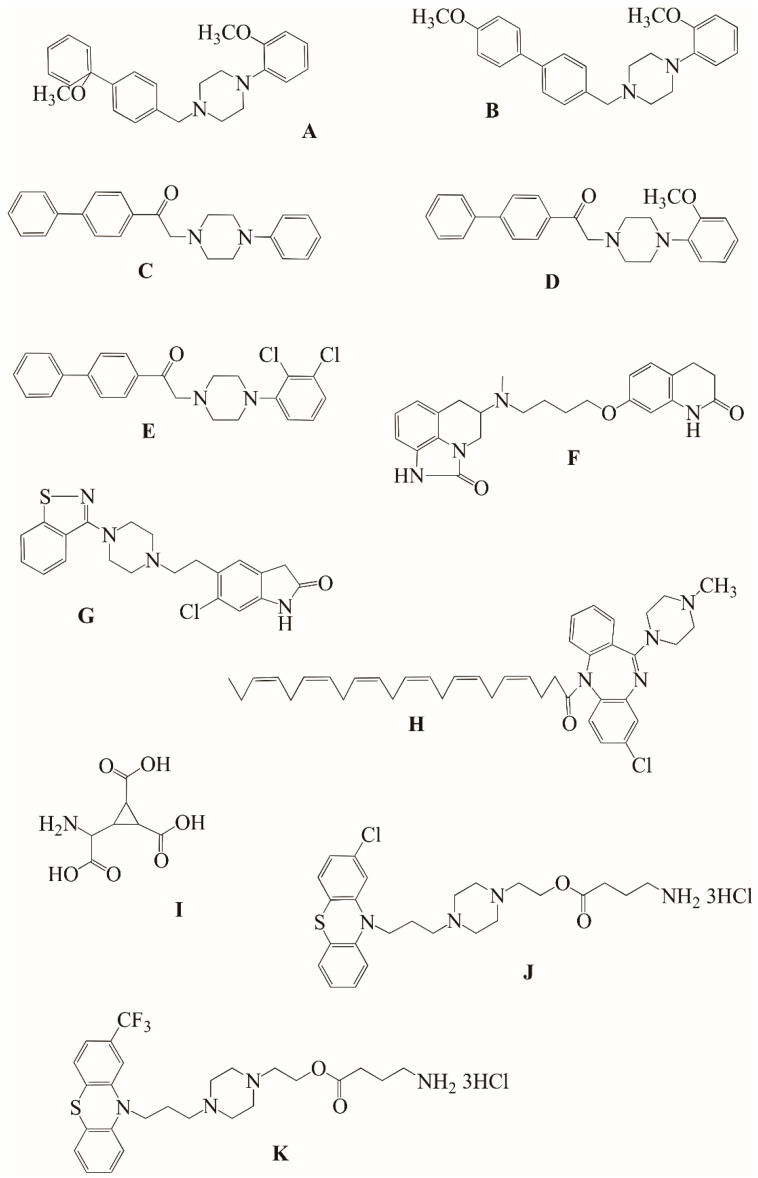
Structures of designed hybrid compounds with anti-schizophrenic properties. (**A**–**E**) Series of conjugates containing the biphenyl pharmacophore and arylpiperazine-like pharmacophore; (**F**) sumanirole–aripiprazole hybrid; (**G**) ziprasidone; (**H**) clozapine–docosahexaenoic acid; (**I**) DCG-IV; (**J**,**K**) GABA-based conjugates (AN168 and AN187, respectively).

Considering the required antagonism toward dopaminergic D2 receptors and 5HT_2_, as well as, for instance, the inflammatory concept of schizophrenia, several hybrids were designed and developed. Indeed, Bhosale et al. [63] as well as Kim et al. [64] proposed a series of hybrids that contain biphenyl moiety of Bifeprunox (1-(2-oxo-benzoxazolin-7-yl)-4-(3-biphenyl)methylpiperazine), linked/fused with modified (modification made on aryl ring) arylpiperazine derivatives. While biphenyl was reported to possess dual D2 and 5HT_1A_ partial agonist activity [65], but also anti-inflammatory activity, arylpiperazine additionally exhibits antidepressant-like effects (Figure 5A–E).

Other in vitro potent candidates for hybrid antipsychotic agents were conjugates of sumanirole, a dopamine D2 receptor full agonist originally developed for the treatment of Parkinson’s disease, substituted at *N*-1 and/or *N*-5 with aripiprazole (Figure 5F) [66]. Although these compounds were not tested in vivo yet, the performed structure–activity relationship (SAR) studies revealed the importance of the sumanirole-substituted region in order to obtain structures with high affinity and enhanced potencies towards dopaminergic D2 receptors.

One of the most intriguing antipsychotics approved by the FDA is ziprasidone (CP-88059; Figure 5G), with a structure based on two diverse, modified, and fused moieties being either DA or a 5HT_2_ ligand. Indeed, this combination resulted in inverse agonist activity at the serotonin 5HT_2A_ receptors, high-affinity antagonist activity at the dopamine D2 receptors, agonist activity at the 5HT_lA_ receptors, and a relatively high affinity for both the serotonin and norepinephrine transporters [67]. The multi-receptor targeting led to ziprasidone being effective in schizophrenia as well as schizoaffective disorders. Moreover, some desired beneficial actions towards negative symptoms and symptoms of depression have been shown, with much fewer side effects in comparison with conventional antipsychotics [68,69].

Since clozapine, the first multi-receptor atypical antipsychotic drug, is superior to neuroleptics for improving psychotic symptoms, but with limited ability to pass through physiological membranes when given orally, significant work was done to efficiently deliver the drug to the appropriate region of the brain, in particular, to cross the BBB. In line with this, fatty acids (FA), and, thus, FA-based conjugates, were proposed to be easily transported across the BBB. Consequently, clozapine and docosahexaenoic acid (DHA, C_22:6-3_) and other conjugates were synthesized and evaluated for their antipsychotic activity [70]. Interestingly, DHA-clozapine (Figure 5H) was much longer acting than clozapine alone, in that the effect of intraperitoneal doses of DHA-clozapine of 3 mg/kg, persisted for 24 h after administration, while in the case of clozapine this was 2 or 4 h.

With regard to the glutamatergic and NMDA hypothesis, several hybrids were designed and their anti-schizophrenic activity was evaluated. DCG-IV (Figure 5I), a C3′-substituted carboxycyclopropylglycine, encompassing the selective metabotropic Glu agonist at mGluR2 and mGluR4 (i.e., L-CCG-I) and an NMDA-selective cyclopropane agonist (i.e., L-CCG-IV), is a perfect example of this [71,72]. This novel structure was reported to depress PCP-induced hyperlocomotion and stereotyped behavior score in mice [73], and the effect was much stronger than that observed for its building element LCCG-I.

The hybridization of both antipsychotic agents and a GABA agonist (including GABA itself), either fused or covalently bound via a carboxylic acid ester bond, also affords new molecules that were found to provide therapeutic effects associated with GABA systems (e.g., mood stabilization and relaxation). In addition, a reduction of side effects induced by popular antipsychotic drugs was reported [74]. In fact, perphenazine and GABA-containing conjugate (codenamed AN168) eliminate catalepsy. A similar effect was reported for fluphenazine and GABA-containing conjugate (AN187), which also significantly reduced catalepsy (Figure 5J,K).

### 2.2. Mood-Related Disorders

Mood (affective) disorders constitute conditions that impact our mood, and thus our functioning. According to the DSM-5, this large group of disorders can now be distinguished into bipolar disorder and depressive disorders; the latter includes a variety of types of depression (from mild to severe).

Mood disorders are known to be characterized by complex pathophysiology with heterogeneous etiologies. Indeed, similarly to previously described schizophrenia and also for mood-related disorders, several possible contributing factors may be distinguished. For instance, it is thought that increased inflammation may be associated with the disease process and contribute to discreet symptomologies in a subset of patients. In fact, when compared to healthy subjects, increased concentrations of pro-inflammatory cytokines, in particular IL-6, have been consistently reported in the blood of patients suffering from either major depression or bipolar disorders [75,76]. The involvement of pro-inflammatory mediators was further supported based on the treatment using NSAIDs, as these (i.e., celecoxib) were found to effectively attenuate depressive symptoms [77].

Nevertheless, the hypothesis of mood-related disorders is not limited to the inflammatory process. Substance-use disorders (SUD) have been long attributed to this type of psychiatric illness. This is true both for alcohol- and drug-use disorders (e.g., cocaine, amphetamines). Intriguingly, chronic use of CNS stimulants generates symptoms that mimic bipolar spectrum disorders, such as euphoria, increased energy, decreased appetite, and grandiosity, as well as paranoia. On the other hand, several reports indicated that some psychiatric effects might be strictly associated with the level of circulating peptide hormones. Indeed, in a paper by Hantsoo and Epperson [78], neurosteroids were shown to be a key component in the premenstrual dysphoric disorder (PMDD) pathogenesis, which is characterized by cognitive-affective symptoms. These symptoms include irritability, depressed mood, anxiety, or mood swings. Importantly, mood symptoms are only present for a specific period, during the luteal phase of the menstrual cycle, as they are relieved in the follicular cycle phase and when hormonal cycling is interrupted [79].

#### 2.2.1. Depression

Depression (also called major depressive disorder, MDD) remains one of the most common states affecting our mind, and can often lead to suicidal thoughts. This type of disorder is not a sign of “weakness of character”, but a serious illness affecting people of all races around the world, of all ages, with different levels of education. The appearance of depression is often linked with several environmental risk factors. These include abnormalities (hyper- or hypothyroidism), cancers (e.g., pancreatic adenocarcinoma and breast cancers), and drug-induced adverse effects (e.g., alpha and beta interferons, isotretinoin, anti-obesity drugs) [80,81,82]. However, a stressful life has become the most popular key driver associated with the onset of depression [83]. In fact, a large body of literature indicates a relationship between emotional and cognitive processes. Mood changes and intense emotions affect attention and memory. The neuroanatomical basis of these conditions is the neural circuits connecting the limbic system and the cerebral cortex.

Since brain areas such as the cortical (the dorsal and medial prefrontal cortex, the dorsal and ventral anterior cingulate cortex, the orbital frontal cortex, and the insula), subcortical (amygdala, hippocampus, and the dorsomedial thalamus), and basal ganglia as well as the brain stem regions are implicated in depression [84], various neurotransmitters are also found in these areas, and are thought to be abnormal in this exact state. Therefore, several neurotransmitter-based hypotheses have been revealed.

***Serotonin hypothesis of depression***

Serotonin plays a crucial role in the development and occurrence of depressive symptoms. The major predictions of this hypothesis are changes in tryptophan metabolism (serotonin synthesis precursor), decreased serotonin level, or disturbed release. Inflammation may also play a role in limiting plasma tryptophan, and thus serotonin activity in the brain [85,86,87]. Of note, “dietary factors” that influence the blood levels of tryptophan and other amino acids can also modify tryptophan uptake in the brain, and consequently the rate of serotonin formation [88].

It has been found that some inhibitory serotonergic receptors, particularly 5HT_1A_, can be divided into two major region-specific populations in the nervous system (i.e., heteroreceptors and autoreceptors, respectively). The postsynaptic heteroreceptors mediate a hyperpolarizing response to released serotonin on pyramidal neurons [89], thus reducing postsynaptic neuronal excitability and firing rates. On the other hand, the presynaptic 5HT_1A_ autoreceptors work in opposition to the abovementioned heteroreceptors, leading to pro-depressive effects [90]. Importantly, this distinct activity revealed by 5HT_1A_ receptors was proposed to serve as a possible mechanism responsible for the delayed efficacy of antidepressants (serotonin selective reuptake inhibitors (SSRIs)).

Apart from 5HT_1A_ receptors, other serotonin receptors, such as 5HT_1B_, were found to play a significant role in depression. Indeed, in humans, reduced 5HT_1B_ function is associated with MDD [91]. This was further supported by several clinical studies showing that 5HT_1B_ receptor agonists produce antidepressant effects in humans. However, similarly to 5HT_1A_, this type of receptor exists in two subpopulations, where a pro-depressive role for the activation of 5HT_1B_ autoreceptors is strongly suggested [92].

Despite substantial evidence of serotonin receptor-specific involvement in depression and depressive-like symptoms, the major pharmaceutical strategy is based on serotonin reuptake inhibitors.

***Dopamine, norepinephrine, and inflammatory-based theory***

Since anhedonia is one of the major symptoms of depression according to DSM-5, the DA system may be strongly involved in the development and occurrence of this type of disorder. Indeed, anhedonia, characterized by an absence of, or decreased ability to experience, pleasure and feel interested, has been linked to a reduction of DA transmission [93]. In line with this, reducing dopaminergic functioning has an adverse effect on the motivation to pursue and work for rewarding stimuli.

However, other neurotransmitters have also been related to reward. This includes opioids, GABA, cannabinoids, which appear to be critical in the experience of consummatory pleasure (“liking”) [94], or serotonin, suggested to be associated with increased impulsivity and preference for immediate reward [95]. Therefore, currently, anhedonia is conceptualized as two diverse states: hedonic vs. motivational [96].

Intriguingly, considering the dopaminergic hypothesis, a novel one was born. A wealth of data has established that DA acts as a major regulator of immune cell function within the brain, as the elevation of cytokines has been found to downregulate processes that govern pre-synaptic dopamine availability and function [97]. This nicely contributes to the results from several meta-analyses of clinical studies showing higher blood concentrations of pro-inflammatory cytokines, including IL-1, IL-6, and tumor necrosis factor (TNF)-α, in depressed patients compared with controls [98,99].

On the other hand, post-mortem and functional imaging studies in the prefrontal cortex of depressed suicide victims revealed the altered density and sensitivity of α_2A_-adrenoceptors, which are known to modulate norepinephrine release, but also norepinephrine’s impact on 5HT neurotransmission [100]. Indeed, a reduced level of norepinephrine resulted in a diminution of voluntary and involuntary motor behaviors, and other catecholamines were first observed in the mid-1950s in patients given compounds that deplete amines (i.e., reserpine, tetrabenazine) [101]. A different set of data implicating norepinephrine systems and α_2A_-adrenoceptors has been provided from pharmacological perturbation studies with the use of clonidine. Clonidine is known for its remarkable stimulation (increase) of growth hormone (GH) concentration in normal healthy subjects. However, in depressive patients, a blunted GH response to this drug was reported [102], thus strongly supporting the norepinephrine hypothesis of depression.

##### Clinically Available Anti-Depressive Medications

Although there are several effective medications on the market, most antidepressants act slowly and may generate a temporary worsening of depression. Nevertheless, drugs that initially proved to be efficacious in depressive disorders were tricyclic antidepressants (TCAs) and monoamine oxidase inhibitors (MAOIs). Unfortunately, they (e.g., clomipramine, amitriptyline) were also characterized as possessing several adverse effects, in particular associated with their non-selective interactions with both serotoninergic and noradrenergic systems. Therefore, a novel class of drug was proposed based on serotonin, which includes selective serotonin reuptake inhibitors (SSRIs), such as citalopram, escitalopram, fluoxetine, fluvoxamine, paroxetine, and sertraline. Again, based on some findings from meta-analyses as well as from randomized controlled trials, these drugs were found ineffective, and a considerable portion of patients suffering from MDD fail to respond adequately to SSRIs [103,104]. In addition, drug–placebo differences in antidepressant efficacy were small [105]. Moreover, it is known that the repeated use of SSRIs can result in an overdose with serious and troublesome side effects, such as gastrointestinal difficulties, weight gain, and sexual dysfunction. Thus, based on the lack of efficacy of SSRIs and the hypothesis of norepinephrine involvement, non-SSRI new-generation antidepressants (NGAs) were developed. This wide group of drugs encompasses serotonin and norepinephrine reuptake inhibitors (SNRI), serotonin modulators and stimulators (SMS), and serotonin antagonists and reuptake inhibitors (SARI), as well as triple reuptake inhibitors (TRIs) that target serotonin (SERT), norepinephrine (NET), and DA (DAT) transporters simultaneously. Of these, only the TRIs revealed their advantages over typical SSRIs or SNRIs. In animal models, such compounds (i.e., a racemic analog of venlafaxine—PRC025 or DOV216303) exhibited antidepressant-like characteristics equal to imipramine [106]. Nonetheless, other safe and potent drug candidates are currently being researched.

##### Hybrid Molecules with Anti-Depressive Potency

Unfortunately, there is scarce information about new compounds designed and developed for depression treatment, possibly due to difficulties related to the disorder’s etiopathology. However, quite recently, therapies targeting genes linked to antidepressant responses have been widely discussed and developed. These include SERT, which appears the most promising, but also DAT, monoamine oxidase (MAO), phosphodiesterase (PDE4), NET, adrenalin_2A_ receptor (ADRA2A), and others.

One of the aforementioned therapies was proposed by Ferrés-Coy et al. [107] and comprised sertraline as a ligand and small interfering RNAs (siRNAs) designed to silence the targeted gene expression, codenamed C-SERT-siRNA. Intriguingly, the intranasal administration of such a combination resulted in increased 5HT signaling and reversion of the depressive-like symptoms in mice treated with corticosterone. The inability to clinically use antidepressants (particularly SSRIs) to discriminate between 5HT_1A_ autoreceptors and postsynaptic 5HT_1A_ receptor results in failed or inadequate responses. The sertraline-based hybrid was also demonstrated to selectively reduce the expression of 5HT_1A_ autoreceptors in the dorsal raphe, without affecting postsynaptic 5HT_1A_ receptor expression [108].

Another example of potent bivalent antidepressants is a group of compounds provided by Cerda-Cavieres et al. [109]. According to the recent knowledge, they synthesized potent SERT ligands: indolyl propyl-piperazine derivatives, which were further merged either with MAOI modulators being morpholine- or D2 receptor modulator-benzoxazinone units, respectively (e.g., compound 7n) [109]. Similarly, Accenta Pharmaceuticals has utilized a hybrid approach to combine the key pharmacophoric moieties of Nocaine (DAT/NET selective) and Modafinil (DAT preferred) to develop the TRI JZ-IV-10, which was characterized by improvements in both the potency and affinity for all the three transporters [110].

**Figure 6:**
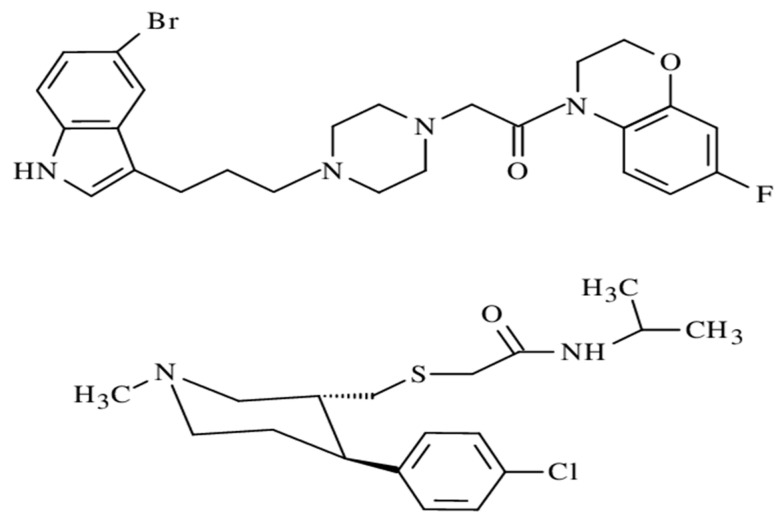
Structures of designed antidepressant hybrids: JZ-IV-10 (**lower structure**) and 7n (**upper structure**).

The structures of all of the aforementioned compounds are presented in Figure 6.

The recent paper by Ortega [111] has shown the anti-depressive action of both galanin and substance P (SP). Consequently, four chimeric structures were synthesized with a galanin sequence at the N-terminus and modified SP located at the C-terminal part of the chain. However, there is still no data regarding its anti-depressive activity in vivo.

Importantly, the studies of Chenu et al. [112] demonstrated that GR205171, a selective and brain penetrant neurokinin NK1 receptor antagonist, selectively potentiated the antidepressant activity of sub-active doses of two SSRIs, citalopram and paroxetine, in the mouse forced-swimming test. Such a discovery prompted other scientists to search for hybrid molecules combining SP ligands with SSRIs. Indeed, Wu et al. [113] and Ryckmans et al. [114] introduced several ligands combining the modified structures of non-peptide SP antagonists (e.g., 4-((3,5-bis(trifluoromethyl)-benzyloxy)-methyl)-4-phenylpiperidine with the 3,5-bis(trifluoro-methyl)benzyl ether side chain appeared to be optimal for NK1 activity) and SSRIs (e.g., modified paroxetine structure). Nonetheless, further studies involving animal models of depression are highly desired.

## 3. Conclusions

As presented in the paper, a significant amount of effort has been made in both academia and industry to resolve the problems associated with the treatment of multifactorial diseases. All investigated bivalent, hybrid molecules have a great potency and potential to act at various biological targets. Nevertheless, although the hybrid approach remains an extremely promiscuous and powerful tool, such novel structures may not to be so-called “master key compounds”, as they can still hit off-target points, resulting in undesirable side effects. It is assumed that adverse effects may be caused by either the drug’s single structural pharmacophores or the hybrid itself. Additional studies, especially in vivo, need to be provided to in order to indicate the therapeutic efficacy of the presented structures.

## Figures and Tables

**Table 1:** Multitargeting of clinically available antipsychotic (neuroleptic) drugs. Here, multitargeting refers to interaction with various and different receptor targets (function of the molecule) not related specifically to the molecule structure [61,62].

Antipsychotic Drug		Drug Targets and Affinities (ki, nm) of Antipsychotics towards Receptor of Interest								
		D1	D2	D3	5HT_2A_	5HT_2C_	H1	MUSCARINIC	α1	α2
***Typical antipsychotics***	Haloperidol 4-[4-(*p*-chlorophenyl)-4-hydroxypiperidino]-4′-fluorobutyrophenone	270	1.4	21	25	>5000	730	>4000	19	>5000
	Chlorpromazine 2-chloro-10-(3-dimethylaminopropy) phenothiazine	6.3	11	9.7	840	0.41	25	1.5	1.4	110
***Atyypical antipsychotics***	Olanzapine 2-methyl-4-(4-methyl-1-piperazinyl)-10H-thieno [2,3-b] [1,5]benzodiazepine	250	17	54	1.9	7.1	3.5	26	60	230
	Clozapine 3-chloro-6-(4-methylpiperazin-1-yl)-11*H*-benzo[b][1,4] benzodiazepine	540	150	360	3.3	13	2.1	34	23	160
	Risperidone 3-[2-[4-(6-fluoro-1,2- benzisoxazol-3-yl)-1-piperidinyl]ethyl]-6,7,8,9-tetrahydro-2-methyl-4H-pyrido [1,2-a]pyrimidin-4-one	620	3.3	13	0.16	63	2.6	>5000	2.3	7.5
	Quetiapine 2-[2-(4-benzo[b][1,4] benzothiazepin-6-ylpiperazin-1-yl) ethoxy]ethanol	>4000	310	650	120	3820	19	1000	58	87

## Abbreviations

Ach, acetylcholine; BBB, blood–brain barrier; CNS, central nervous system; DA, dopamine; DAT, dopamine transporter; DHA, docosahexaenoic acid; DMLs, designed multiple ligands; DSM-5, Diagnostic and Statistical Manual of Mental Disorders; EPS, extrapyramidal symptoms, FDA, Food and Drug Administration; GH, growth hormone; Glu, glutamate; MAO, monoamine oxidase; MAOIs, monoamine oxidase inhibitors; MDD, major depressive disorder; NET, norepinephrine transporter; NK-1, neurokinin receptor 1; NMDA, *N*-methyl-D-aspartate; NSAIDs, nonsteroidal anti-inflammatory drugs; OCD, obsessive–compulsive disorder; PANSS, positive symptoms and general psychopathology; PCP, phencyclidine; PFC, prefrontal cortex; PMDD, premenstrual dysphoric disorder; SERT, serotonin transporter; SNRIs, selective serotonin and norepinephrine reuptake inhibitors; SP, substance P; SSRI, selective serotonin reuptake inhibitors; SUD, substance-used disorder; TCA, tricyclic antidepressant; TRI, triple reuptake inhibitors.
